# YOLO-IHD: Improved Real-Time Human Detection System for Indoor Drones

**DOI:** 10.3390/s24030922

**Published:** 2024-01-31

**Authors:** Gokhan Kucukayan, Hacer Karacan

**Affiliations:** 1 Informatics Institute, Gazi University, 06680 Ankara, Turkey; 2 Computer Engineering Department, Faculty of Engineering, Gazi University, 06570 Ankara, Turkey; hkaracan@gazi.edu.tr

**Keywords:** UAV, indoor drone, real-time object detection, YOLO-IHD

## Abstract

In the field of unmanned systems, the combination of artificial intelligence with self-operating functionalities is becoming increasingly important. This study introduces a new method for autonomously detecting humans in indoor environments using unmanned aerial vehicles, utilizing the advanced techniques of a deep learning framework commonly known as “You Only Look Once” (YOLO). The key contribution of this research is the development of a new model (YOLO-IHD), specifically designed for human detection in indoor using drones. This model is created using a unique dataset gathered from aerial vehicle footage in various indoor environments. It significantly improves the accuracy of detecting people in these complex environments. The model achieves a notable advancement in autonomous monitoring and search-and-rescue operations, highlighting its importance for tasks that require precise human detection. The improved performance of the new model is due to its optimized convolutional layers and an attention mechanism that process complex visual data from indoor environments. This results in more dependable operation in critical situations like disaster response and indoor rescue missions. Moreover, when combined with an accelerating processing library, the model shows enhanced real-time detection capabilities and operates effectively in a real-world environment with a custom designed indoor drone. This research lays the groundwork for future enhancements designed to significantly increase the model’s accuracy and the reliability of indoor human detection in real-time drone applications.

## 1. Introduction

In the rapidly advancing field of unmanned aerial vehicle (UAV) technology, object detection remains a pivotal challenge, especially in the context of indoor environments. Indoor scenarios pose unique difficulties for UAVs, including constrained spaces, varied lighting conditions, and complex backgrounds, making effective human detection a task of critical importance. While current advancements in deep learning have provided substantial progress in this domain, there is a significant need to tailor these technologies to suit the intricacies of indoor surveillance and navigation.

In recent years, the integration of unmanned aerial vehicles (UAVs) in surveillance and monitoring tasks has catalyzed the evolution of human detection systems. While vision-based systems, using algorithms like deep learning models, have been the cornerstone of UAV-based human detection, alternative approaches are emerging, addressing the limitations of purely visual techniques. Radar-spectrogram analysis, for instance, utilizes deep learning models to interpret micro-Doppler signatures of targets, presenting a novel method for identifying human activities from UAVs in real-time scenarios [1]. Alternatively, recent studies in sensors are increasingly leveraging a variety of sensor modalities to enhance detection accuracy, particularly in challenging environments like indoor or obscured environments. Thermal imaging and ultrawideband (UWB) sensing technologies offer significant advancements in detecting humans, even in conditions where visual systems cannot succeed. UWB sensing, specifically, has shown remarkable capabilities in distinguishing between drone and human movements in confined indoor spaces, demonstrating the effectiveness of multi-modal sensing strategies in UAV-based surveillance [2]. These advancements in integrating diverse data sources, such as air quality monitoring and heat source detection, not only improve detection precision but also pave the way for UAV applications in more complex and dynamic environments. The use of these alternative technologies underscores a significant shift in UAV surveillance methodology, broadening the spectrum of applications and offering more robust solutions for real-time human detection in various operational scenarios. In addition to vision-based systems, alternative approaches for real-time human detection are leveraging various data sources such as air quality monitoring and heat source detection. Thermal imaging-based systems have become crucial in smart video surveillance for moving human detection in thermal videos, even in low-light or cluttered backgrounds. This technology captures heat generated from humans, offering a vital solution for safety and security by minimizing crime and trespassing through enhanced identification and monitoring [3]. Furthermore, the integration of Internet of Things (IoT) sensor grids in households with multiple heating systems has opened new avenues for air contaminant migration monitoring. This approach offers continuous monitoring with data transfer to the cloud, enabling the near-real-time detection of unscheduled or unauthorized access to specific areas. The utilization of such technology in UAVs could transform surveillance capabilities, allowing for the dynamic measurement of contaminants and providing real-time access control. This novel application of IoT, in line with the Industry 4.0 concept, allows for extensive data analysis over longer periods, enabling predictions of occupant behavior or the need for ventilation in specific rooms or areas, thus enhancing the capabilities of UAVs in complex surveillance scenarios [4].

Small-object detection, a crucial aspect of UAV image processing, aims to identify objects that are small, complex, and challenging to distinguish by color. Traditional detection methods, which depend on manually designed features, encounter significant challenges in UAV-based applications. These challenges include sensitivity to varying lighting conditions, angles, and obstructions, and difficulties in processing complex backgrounds. While effective in simpler environments, these methods often lead to false positives and missed detections in more intricate scenarios [5,6,7]. Conversely, the advent of deep learning, especially convolutional neural networks (CNNs), has markedly improved the detection of small-scale objects in UAV imagery, addressing many limitations inherent in traditional techniques [8,9].

In deep learning for UAV small-object detection, the algorithms are primarily divided into two categories: two-stage and single-stage detectors. Two-stage detectors, including algorithms like R-CNN [10], Faster R-CNN [11], Mask R-CNN [12], and Cascade R-CNN [13], are recognized for their higher accuracy. They are particularly effective in detecting small objects against complex backgrounds, employing a process that initially generates proposal regions and subsequently performs classification and regression on these regions. However, this method is computationally intensive, leading to slower processing speeds. In contrast, single-stage detectors such as the YOLO series [14], SSD [15], RetinaNet [16], and CenterNet [17] are known for their rapid processing speeds and real-time performance capabilities. However, these models tend to have lower accuracy in detecting small targets within complex backgrounds, which can result in false positives or missed detections. The trade-offs between these two approaches underscore the ongoing challenges and developments in UAV image processing, emphasizing the need for a balance between accuracy, speed, and computational efficiency. Han et al. [18] introduced the DRFBNet300, a lightweight single-stage method achieving high accuracy and real-time performance in UAV imagery. Additionally, Zhang et al. [19] proposed the DAGN, a YOLOv3 based model, which improved detection accuracy while maintaining real-time detection capabilities. These studies show the advancements and ongoing research in UAV-based object detection systems.

While the performance of general object detectors has been commendable, their application in indoor human detection using drones necessitates addressing specific challenges unique to this context. Unlike outdoor, indoor environments are characterized by varying lighting conditions, potential obstructions like furniture, and confined spaces, all of which can severely affect the quality of detection. In the realm of indoor drone operations, objects are often captured from diverse perspectives and at varying distances. This variability can be more pronounced indoors due to the limited space and the drone’s proximity to objects. As a result, performing object detection at a consistent scale becomes challenging, often leading to significant errors and missed detections. This is especially critical in scenarios where human detection is paramount, such as search-and-rescue operations or security surveillance.

Moreover, indoor scenes frequently contain densely packed elements and small-scale features. Humans might be partially or heavily occluded by furniture or other indoor structures, making the distinct features necessary for accurate detection less discernible. These challenges are compounded by the tendency of drone images to lose detail due to down sampling during detection. In indoor environment, this can mean the critical loss of features necessary for identifying and distinguishing humans, especially in crowded or complex scenes.

Considering these factors, current detection methods often struggle with precisely detecting humans in the indoor drone’s line of sight. Due to these reasons, this study aims to develop a human detection model that detects in real-time on an onboard computer equipped within a drone, demonstrating resilience to indoor environmental conditions.

This paper focuses on adapting and enhancing the YOLOv7 architecture, a state-of-the-art object detection model, to better suit indoor scenarios. YOLOv7’s exceptional balance of speed and accuracy makes it a suitable candidate for real-time applications; however, its standard implementation is primarily oriented towards outdoor or generic settings. Recognizing this, our research aims to modify and optimize YOLOv7-tiny, creating a variant specifically tuned for the challenges of indoor UAV operations. This involves customizing the model to better handle the diverse range of indoor conditions and to improve its efficiency in accurately detecting humans in such environments.

The proposed methodology of this research is twofold. First, we adapt the YOLOv7-tiny model through a long process of retraining and fine-tuning, using a dataset specifically curated for indoor UAV scenarios. This dataset comprises a diverse range of UAV perspective images and indoor human environments, encompassing various lighting conditions, room sizes, and clutter levels to ensure comprehensive learning. Second, we introduce modifications to the YOLOv7-tiny architecture to enhance its ability to detect humans in indoor settings. These modifications include optimizing the model’s convolutional layers, integrating enhanced spatial pyramid pooling (SPP), and implementing a more robust activation function to improve network performance to reduce false positives and improve detection accuracy in confined spaces.

The main contributions of this paper are as follows:

1. The development of YOLO-IHD, an adaptation of the YOLOv7 architecture specifically tailored for indoor UAV-based human detection, which significantly enhances detection accuracy in complex indoor environments.
2. The proposed model of this paper outperforms the pre-trained YOLOv7-tiny model in terms of average precision, with a 42.51% increase in mAP@0.5 on the IHD dataset, and a 33.05% increase on the VisDrone dataset. This advancement is critical for applications demanding high precision in human detection from drones.
3. The lack of a dataset for indoor human detection by drones led to the creation of the IHD dataset. This new dataset presents a wide range of human images from diverse indoor perspectives, combined with existing, widely utilized datasets, resulting in a unique and comprehensive resource tailored to models specializing in indoor human detection.
4. The optimization of the convolutional layers and attention mechanism in YOLO-IHD, which adeptly process complex visual data from indoor environments, ensuring higher reliability in scenarios such as disaster response and indoor rescue missions.

This paper is structured so as to first provide a detailed overview of related work in indoor drone-based human detection and the evolution of YOLO models. Subsequently, we deep-dive into the methodology, elaborating on the model modifications and customizations, and dataset preparation and training processes. This is followed by a comprehensive presentation of our experimental setup, results, and comparative analysis with existing models. Finally, this paper concludes by discussing the implications of our findings for indoor UAV applications and suggesting directions for future research.

## 2. Related Work

### 2.1. Deep Learning-Based Detection Methods

Deep learning-based detectors can be broadly categorized into two types: two-stage and one-stage detectors. Within the domain of one-stage detectors, the Single Shot Multibox Detector (SSD) [20] and You Only Look Once (YOLO) have been developed to address the balance between accuracy and processing time. Notably, YOLO is acknowledged for effectively managing performance in terms of both accuracy and processing time.

The CNN-based detectors, comprising RCNN [21], Fast RCNN [22], and Faster RCNN [11], belong to the two-stage category, showcasing superior accuracy compared to various other detection algorithms. Nevertheless, these methods are associated with a downside of heightened computational costs, resulting in extended processing durations.

### 2.2. Two-Stage Detectors

In multi-stage detectors, one model is used to extract regions of objects, and the other one is used to classify and further detect the location of the object. R-CNN, which stands for region-based convolutional neural network, is an algorithm designed for object detection and comprises multiple versions. In the initial iteration of R-CNN, a technique called selective search is employed for region proposal, identifying potential areas containing objects. These identified regions undergo resizing and processing through a pre-trained convolutional neural network (CNN) to extract features. Support vector machine (SVM) classifiers are then trained for each object category, and a regression model for bounding boxes refines the localization. Fast R-CNN builds upon this improvement by introducing RoI (region of interest) pooling, eliminating the necessity for region warping. This advancement allows for end-to-end training, making the entire system jointly trainable. Faster R-CNN takes efficiency a step further by incorporating a region proposal network (RPN) into the model. This integration removes the dependence on external region proposal methods, enabling end-to-end training and resulting in a unified framework.

### 2.3. One-Stage Detectors

One-stage detection focus on predicting bounding boxes directly; there is no intermediate task such as region proposals which must be performed in order to produce an output. Therefore, a simpler and faster model architecture is obtained.

Single Shot Multibox Detector (SSD) is an object detection algorithm designed for the efficient and accurate detection of objects in images. Unlike traditional two-stage detectors, SSD is a one-stage detector, aiming to balance speed and accuracy using the concept of “multibox”. Multiboxes incorporate multiple bounding boxes with different aspect ratios and scales for each location in the feature map. Default or anchor boxes at various positions in the feature maps act as initial points for predicting both object bounding boxes and class scores. The model employs a feature pyramid network (FPN) to extract features at multiple scales, thereby improving its capability to detect objects of varying sizes. The prediction of object scores and bounding box coordinates occurs directly from multiple feature maps at different scales within a single forward pass. SSD’s loss function combines classification loss (utilizing softmax) and regression loss (employing smooth L1 loss) to facilitate accurate model training for localization. SSD offers advantages such as the ability to conduct object detection in a single forward pass, handling objects with diverse sizes and shapes through default boxes, and efficient training by simultaneously optimizing classification and localization tasks. It is well suited for real-time applications, including video processing. Nevertheless, SSD involves trade-offs, including potential accuracy compromises compared to more thorough two-stage detectors, and difficulties in detecting small objects, particularly in the presence of larger objects in the same image [23].

The YOLO (You Only Look Once) algorithm represents a significant leap in computer vision for real-time object detection. Unlike conventional approaches, YOLO conducts detection in a single pass through the neural network by dividing the input image into a grid. Each grid cell predicts bounding boxes and class probabilities, allowing the identification of multiple objects within the same cell. The predictions involve simultaneous determination of both direct bounding box coordinates and associated class probabilities. YOLO incorporates multiple scales to enhance object detection efficiency, accommodating objects of various sizes. Following predictions, non-maximum suppression is applied to enhance the precision of the final set of detected objects by removing redundant bounding boxes.

YOLO receives acclaim for its ability to operate in real-time, making it well suited for applications such as video analysis and autonomous vehicles. Its unified framework removes the need for separate region proposal networks, contributing to overall efficiency. YOLO manages to strike a balance between accuracy and speed, making it adaptable for diverse applications. However, YOLO may encounter difficulties in accurately identifying small objects due to the limitations of a single-grid cell. Additionally, there might be a trade-off in localization accuracy compared to two-stage detectors, particularly for objects with intricate shapes. In summary, YOLO has gained prominence for its efficient and effective real-time object detection capabilities across a wide range of applications.

### 2.4. YOLOv7

YOLOv7 [24] represents a significant advancement in the field of real-time object detection, known for its impressive speed and accuracy. Its development is attributed to the collaborative efforts of Wong Kin Yiu and Alexey AB, who have made substantial contributions to the YOLO family of models. YOLOv7 was designed to set new benchmarks in object detection by predicting bounding boxes more accurately and quickly compared to its predecessors and peers. One of the key elements of YOLOv7 is its efficient layer aggregation, which focuses on the convolutional layers in the backbone. This efficiency is critical for fast inference speeds. The model builds on the concept of cross stage partial networks, which was instrumental in making YOLOv4 [25] and YOLOv5 [26] more efficient. The final layer aggregation in YOLOv7, known as E-ELAN (extended–efficient layer aggregation network), is an extended version of the ELAN computational block. This design optimizes both the gradient path length and the stacking number of computational blocks, leading to a network that learns more diverse features effectively. Model scaling is another crucial aspect of YOLOv7. The model considers the depth and width of the network, scaling these attributes in concert while concatenating layers. This compound scaling approach ensures optimal architecture while scaling the model for different sizes, making it adaptable for various applications that require different levels of accuracy and inference speeds. Re-parameterization techniques in YOLOv7 involve averaging a set of model weights to create a more robust model. This approach focuses on module-level re-parameterization, where different parts of the network have their own re-parameterization strategies based on gradient flow propagation paths. The YOLOv7 network also features an innovative auxiliary head concept, referred to as the lead head and coarse-to-fine lead head. The auxiliary head is supervised in addition to the lead head during training, with the former learning coarser and the latter finer details. This deep supervision enables the model to capture more nuanced features and improves overall detection performance. In terms of results, YOLOv7 surpasses previous object detectors in both speed and accuracy, showing significant improvement over a range of 5 FPS to 160 FPS. The model demonstrates its superiority by outperforming earlier YOLO versions and other baseline models in mean average precision (mAP) across various model sizes.

### 2.5. YOLOv7-Tiny Model

**Figure 1:**
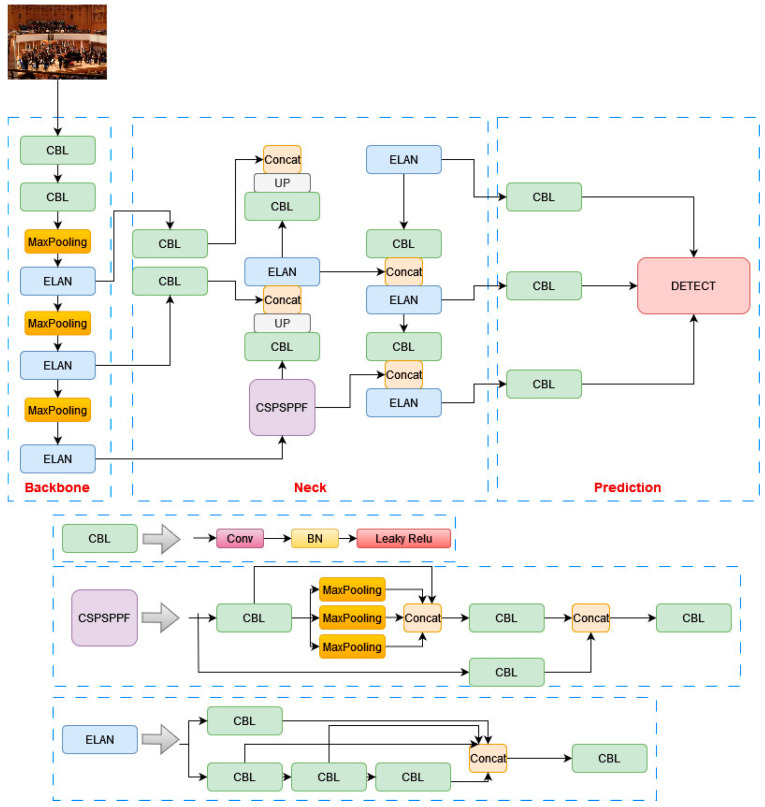
YOLOv7-tiny network structure.

YOLOv7-tiny is an adaptation of the more complex YOLOv7 model, tailored specifically for edge GPUs, which are known for their limited computational resources. In order to make it suitable for such environments, YOLOv7-tiny employs a streamlined architecture, as shown in Figure 1, while maintaining the core components of the YOLOv7 model: the backbone, neck, and head. The backbone of YOLOv7-tiny uses ELAN-T, a simpler version of the extended–efficient layer aggregation network (E-ELAN) found in the full YOLOv7 model. This change includes the removal of the convolution operation in MPConv, relying solely on pooling for down sampling. Despite these reductions, the optimized spatial pyramid pooling (SPP) structure is retained, ensuring that rich feature maps are still provided to the neck layer. This balance between simplification and feature richness is crucial for maintaining effective detection capabilities within the constraints of edge devices. In the neck, YOLOv7-tiny continues to use the PANet structure, a design choice that facilitates efficient feature aggregation from different levels of the backbone. This helps in preserving important information necessary for accurate object detection. At the head of the network, YOLOv7-tiny opts for standard convolution to adjust channel numbers instead of using the more complex REPConv. This modification is part of the model’s strategy to reduce computational load and memory requirements. While YOLOv7-tiny has decreased accuracy compared to its full-sized counterpart, it offers significant advantages in terms of speed and model size, making it well suited for applications where resources are limited and real-time performance is essential. This balance of speed, weight, and accuracy makes YOLOv7-tiny an appealing option for edge computing applications in real-time object detection.

### 2.6. Real-Time Detection

This section encapsulates the state of the art in real-time object detection using indoor drones, examining the technological advancements, the challenges faced, and the future directions of real-time UAV systems. By analyzing recent studies and developments, it aims to offer a comprehensive understanding of how indoor drones are being equipped to perform real-time object detection, their current capabilities, and the potential they hold for future applications. The list of studies is shown in Table 1.

### 2.7. Techniques for Drone Positioning and Attitude

The effectiveness of unmanned aerial vehicles (UAVs) in indoor human detection is significantly influenced by their ability to accurately determine their position and attitude within complex indoor environments. Precise positioning and attitude data are essential for creating a reliable dataset and enabling the fine-grained perception of human activities. These challenges are exacerbated in indoor settings due to the lack of GPS signals and the presence of obstacles affecting UAV navigation and stability. In response, innovative methods have been developed specifically for indoor UAV applications. Cao et al. [49] have enhanced indoor positioning accuracy using a WiFi RTT algorithm, capable of compensating for LOS and identifying NLOS conditions, crucial for UAV stability in indoor environments. Concurrently, Bi et al. [50] introduced a low-cost UAV detection method through WiFi traffic analysis combined with machine learning, offering a novel approach for UAV monitoring in complex environments. Complementing these, Liang et al. [51] proposed an attitude estimation method for quadrotor UAVs based on the quaternion unscented Kalman filter (QUKF), enhancing navigational precision. Furthermore, Cheng et al. [52] addressed GPS-denied environments by proposing a dynamic autonomous docking scheme for UAVs and UGVs, facilitating effective navigation and operation in constrained settings. Integrating these methods into UAV-based human detection systems significantly enhances the reliability and accuracy of data collection. Precise indoor positioning, coupled with effective attitude estimation, enables UAVs to accurately navigate and capture data in challenging indoor environments, proving vital for applications in surveillance, search-and-rescue operations, and detailed human activity monitoring.

Integrating light detection and ranging (LiDAR) and visual odometry for indoor drones has shown significant advancements in improving positioning and attitude determination in GPS-denied environments. Bautista et al. [53] developed a system combining a vision-based photogrammetric position sensor and visual inertial odometry for precise quadcopter landing, while Wang et al. [54] demonstrated a 74.61% improvement in positioning accuracy using a LiDAR-aided integrated navigation system. Additionally, Qiu et al. [55] introduced a LiDAR-inertial navigation system that effectively addresses the challenges in feature extraction within spatial grid structures, enhancing pose estimation accuracy in GNSS-denied settings. These studies collectively highlight the progress and potential of sensor integration in unmanned aerial vehicles for reliable and precise indoor navigation.

Upon a thorough examination of the indoor drone navigation and positioning techniques previously discussed, this study has determined that the most suitable approach for implementation is the combination of LiDAR and visual odometry. This decision is underpinned by the distinct advantages of each technology: LiDAR’s exceptional accuracy in distance measurement and obstacle detection, and visual odometry’s capability in precise position tracking through visual techniques.

Related to this method, for indoor flights and data collection phases, it has been resolved that employing LiDAR for its robust obstacle detection capabilities will greatly enhance the safety and efficiency of the drone’s navigation in complex indoor environments. Concurrently, the integration of visual odometry will provide a reliable method for real-time positioning, ensuring a high degree of accuracy in the drone’s trajectory and spatial orientation. This combination approach not only mitigates the individual limitations of each system but also capitalizes on their combined strengths to offer a comprehensive solution for indoor drone position and attitude.

### 2.8. Security Aspects in UAV Communication Systems

The evolution of drone technology has expanded the capabilities and applications of unmanned aerial vehicles (UAVs), but it also introduces significant security challenges, particularly in communication systems. Drones, especially in complex environments like indoor human detection, require secure and reliable communication channels to function effectively. One of the fundamental concerns is the vulnerability of these channels to various cyber threats. The research by Krichen et al. [56] highlights the susceptibility of drone communications to attacks such as man-in-the-middle, denial-of-service, and data interception. These vulnerabilities can have dire consequences, especially when drones are used in sensitive areas or for critical missions. The authors emphasize the necessity of robust security protocols and propose countermeasures like blockchain technology and machine learning techniques to enhance drone communication security.

The security of drone communication systems is not just limited to preventing unauthorized access or data breaches. It extends to ensuring the integrity and confidentiality of the transmitted data. Ronaldo et al. [57] discuss the implementation of a Forward Prediction Scheduling-based Stream Control Transmission Protocol (FPS-SCTP) in drones, which offers an enhanced real-time data transmission capability while ensuring robust security through encryption mechanisms like AES and digital signatures with ECDSA. This approach is particularly beneficial in environments where drones are used for delivery services or situations requiring immediate data transmission with high security.

Moreover, Ko et al. [58] address the pressing need for secure UAV-to-UAV communication, emphasizing the importance of protocols that provide non-repudiation and perfect forward secrecy, especially in military settings. They propose a security protocol with two sub-protocols to secure communication between UAVs and between a UAV and a ground control station, achieving essential security requirements such as confidentiality, integrity, mutual authentication, non-repudiation, and resilience against various attacks, including DoS and man-in-the-middle attacks.

In conclusion, the security of drone communication systems is crucial in ensuring their effective and safe operation. Advanced encryption techniques and innovative protocols like FPS-SCTP are essential in safeguarding these systems against a wide range of cyber threats, thus enhancing the overall reliability and functionality of drones in various applications.

### 2.9. Ethical and Privacy Considerations in Indoor Surveillance by Autonomous Drones

In this study, the introduction of the proposed model, aimed at real-time human detection in indoor environments using autonomous drones, is presented. The focus extends beyond technical enhancements, encompassing the broader implications of its application in the context of ethical, privacy, and societal impacts. The importance of balancing technological advancement with the preservation of human rights is underscored, highlighting the need to take into account ethical considerations, privacy concerns, regulatory compliance, and public engagement in the deployment of such surveillance technologies.

The integration of drones for surveillance, particularly in indoor settings, raises significant ethical considerations. The primary concern revolves around the balance between enhancing security and respecting individual privacy rights. It is imperative to ensure that such technology is employed responsibly, adhering to legal and ethical standards. Moreover, the deployment of surveillance drones necessitates compliance with local and international privacy regulations. This includes securing necessary permissions and consents, especially when operating in private indoor spaces. These steps are crucial to maintain public trust and the legitimacy of using drones for security purposes [59].

Privacy implications of indoor drone surveillance are a paramount concern. Effective measures, such as data anonymization and secure data storage protocols, must be implemented to safeguard individual privacy. Additionally, transparency in the operational use of drones is essential. Clear policies should be established outlining who has access to the data, their intended use, and accountability mechanisms. Engaging the public through education about the technology’s benefits, limitations, and the measures taken to uphold privacy and ethical standards is also vital for gaining public acceptance and trust [60].

Continuing with the theme of ethical considerations and privacy implications, another important aspect to consider is the impact of autonomous surveillance drones on societal norms and expectations of privacy. The use of drones for human detection in indoor environments challenges traditional notions of privacy, necessitating a reevaluation of what constitutes reasonable expectations in the age of advanced surveillance technologies. It is crucial to engage in an ongoing dialogue with stakeholders, including policymakers, technologists, and the public, to establish norms and guidelines that respect individual rights while leveraging the benefits of drone technology. This dialogue should aim to create a consensus on the acceptable use of such technologies, ensuring that their deployment does not infringe on fundamental privacy rights and civil liberties [61,62].

## 3. Methodology

This section outlines the comprehensive approach taken to adapt and enhance the YOLOv7-tiny model for improved human detection in indoor UAV scenarios. The methodology is structured into four main components and each component is designed to address specific challenges associated with indoor environments. These are as follows:

1. Adding a Small-Object Detection Layer: The first step in the methodology focuses on enhancing the model’s ability to detect small objects, a common challenge in object detection tasks. This involves integrating a specialized layer dedicated to small-object detection. The addition of this layer aims to address the limitations of standard convolutional layers in capturing the finer details of smaller objects, which are often lost due to spatial pooling and lower resolution feature maps. The layer will operate at a higher resolution, enabling the model to better identify and classify smaller objects that are crucial in diverse detection scenarios, particularly in environments where such objects are of specific interest.
2. Implementing CSPSPPF (cross stage partial spatial pyramid pooling—fast): The second component of the methodology is the integration of the CSPSPPF module, a novel variation of the traditional spatial pyramid pooling framework. This module combines the benefits of cross stage partial (CSP) architecture and spatial pyramid pooling (SPP), further optimized for speed. The CSP structure reduces computational redundancy and improves the efficiency of feature map utilization, while the SPP component ensures robust multi-scale feature aggregation. The ‘Fast’ aspect of CSPSPPF indicates a streamlined approach, focusing on maintaining high processing speeds, making the model suitable for real-time applications where both accuracy and efficiency are critical.
3. Adopting the Mish Activation Function: The final aspect of the methodology involves the implementation of the Mish activation function within the network. Mish is known for its smooth, non-monotonic behavior, which has been shown to facilitate better gradient flow compared to traditional activation functions like ReLU. This can lead to improved learning dynamics, allowing the network to capture more complex patterns and nuances in the data. The use of Mish is particularly beneficial in deep learning models, as it can enhance the model’s overall accuracy and generalization capabilities, making it more effective in diverse and challenging object detection tasks.
4. Data Augmentation for Indoor Scenarios: The fourth step in the methodology emphasizes data augmentation specifically tailored for indoor environments. This involves simulating a range of indoor conditions, such as variable lighting, occlusions, and diverse interior layouts to create a more comprehensive training dataset. Techniques like adjusting brightness and contrast, applying blur to mimic motion or focus variations, and artificially altering backgrounds help in preparing the model for the complexities of indoor detection. This augmentation not only aids in enhancing the model’s robustness against overfitting but also ensures better generalization when deployed in real-world indoor settings.

**Figure 2:**
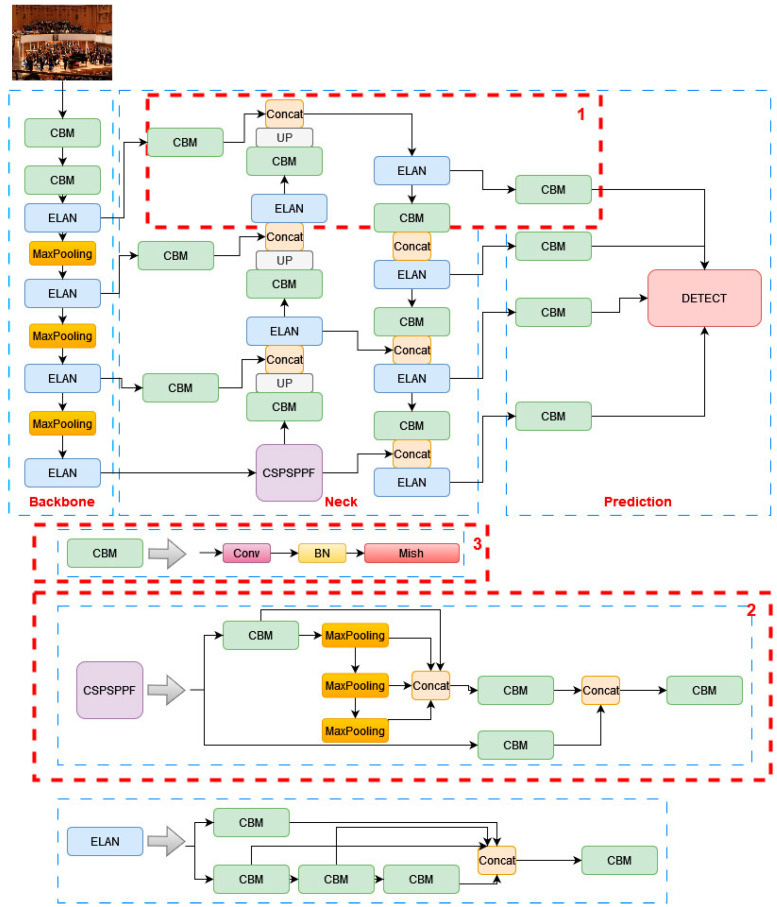
YOLO-IHD network structure. 1. Adding small object detection layer. 2. Chancing to Faster SPP structure. 3. Using Mish activation function.

The network structure of YOLO-IHD is shown in Figure 2, with the improvements highlighted in red striped squares.

### 3.1. Small-Object Detection Layer

Small objects contain fewer pixels and less information compared to larger objects. Therefore, higher resolution feature maps are more effective in preserving the details of small objects. YOLOv7-tiny typically uses feature maps at resolutions of 20 × 20, 40 × 40, and 80 × 80. In the proposed model for indoor human detection using drones, the challenge of detecting small objects is addressed by modifying the detection head. While the YOLOv7-tiny model demonstrates proficiency in various applications, its performance in detecting smaller objects is suppressed by the convolution-based feature extraction mechanism. As the network depth increases, the feature map resolution decreases, leading to potential inaccuracies in small target detection. The original YOLOv7-tiny model comprises three detection heads with resolutions of 20 × 20, 40 × 40, and 80 × 80. A new small-object detection layer is added to the base model. This layer, comparable to the P2 layer in baseline model, has a resolution of 160 × 160, allowing for more detailed features and enabling the detection of smaller targets in complex indoor environments. This layer was integrated into the YOLOv7-tiny model to enhance its ability of detecting human figures in the varied and often cluttered backgrounds typical of indoor environments. This addition not only improved the model’s detection performance for smaller targets but also maintained its accuracy for larger ones. The incorporation of the P2 layer detection head, with its high-resolution feature map, proved especially effective in addressing the challenges posed by scale variance and complex indoor scenarios, such as low illumination and shadow occlusion, which are often encountered indoors. With the addition of the small-object detection layer, the model’s detection architecture now includes heads with resolutions of: 20 × 20, 40 × 40, 80 × 80, and the newly added 160 × 160. This expansion in the detection heads significantly enhances the model’s capacity to accurately identify objects across a broader range of sizes, particularly improving its efficacy in detecting smaller objects in indoor environments.

### 3.2. Spatial Pyramid Pooling (SPP)

The YOLOv7-tiny neural network model integrates the spatial pyramid pooling (SPP) [63] module, which executes pooling operations at various scales. SPP, positioned after the final convolutional layer and shown in Figure 2, aggregates contextual information from diverse regions of the image. This implementation generates multiple representations of feature maps, each utilizing distinct window sizes and strides, to accommodate the varying scales within an image. This multi-scale pooling approach is especially beneficial for the proposed model where the size of human figures can drastically differ within the same scene. For example, in a room scenario, a person nearer to the UAV may appear larger, while someone further away might be smaller. SPP enables the model to effectively capture features relevant to both scenarios, thereby enhancing human detection capabilities at varying distances from the camera. In the YOLOv7-tiny architecture, the SPP module is advanced into the cross stage partial spatial pyramid pooling (CSPSPP) [24]. An evolution of the traditional SPP, CSPSPP incorporates a CSP structure and offers improved performance for the proposed dataset. While CSPSPP significantly enhances feature representation, it also increases the number of parameters and computational load.

Conversely, SPPF (SPP-Fast) [64] optimizes efficiency by replacing the parallel pooling operations in SPP with a serial arrangement. It employs consecutive 5 × 5 pooling layers, where two such layers are equivalent to a single 9 × 9 pooling layer and three to a 13 × 13 layer. This serial methodology not only maintains the effectiveness of parallel pooling but also increases processing efficiency and detection accuracy. This study adopts SPPF to enhance model accuracy and the efficiency of feature fusion in the YOLOv7-tiny model. Due to its reduced computational load, this approach is well suited for real-time detection models on edge devices. The adaptation of CSPSPPF in this context is illustrated in Figure 2.

### 3.3. Activation Function

The adoption of Mish [65] as the activation function in the proposed lightweight model, characterized by a limited number of parameters and calculations, is a strategic decision to enhance the model’s performance without increasing deployment costs. Unlike LeakyReLU [66], which struggles to establish a consistent link between positive and negative input values, Mish offers a more seamless transition, thus facilitating fewer but more effective feature extraction operations. This shift to Mish ensures a smooth gradient flow and a broader range of neuron activation states, crucial for complex pattern detection and classification. It also addresses the risk of inactive neurons better than LeakyReLU, making the learning process more dynamic across the network. Consequently, with Mish, the model not only learns and performs better but also does so with improved efficiency and generalization, as evidenced by empirical studies. These advantages are achieved without compromising on computational efficiency, thus maintaining the lightweight nature of the model. The equations governing Mish and LeakyReLU shown as Equations (1) and (2).

(1) $$\mathrm{LeakyReLU}\left(x\right)=\left\{\begin{array}{ll} x & x>0\\ \alpha x & x\leq 0 \end{array}\right.$$

(2) $$\mathrm{Mish}\left(x\right)=x\cdot \tanh\left(\ln\left(1+e^{x}\right)\right)$$

**Figure 3:**
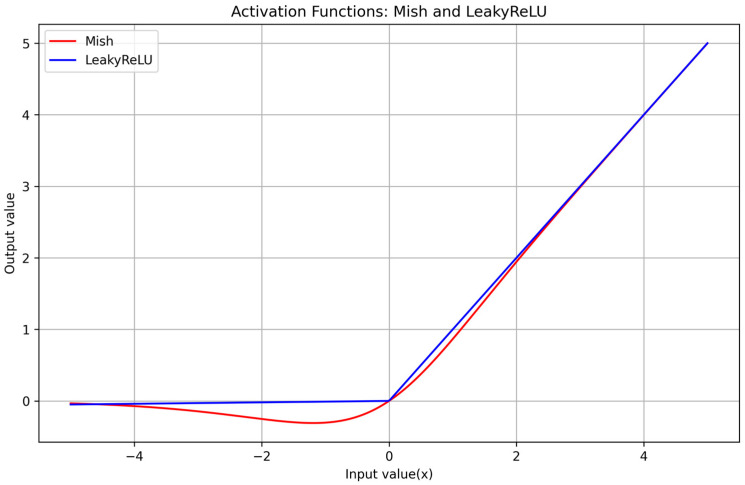
Input/Output value graph of Mish and LeakyReLU activation functions.

In Figure 3, the curve of the Mish function for *x* within the range of [−5, 5] is showcased, illustrating key features of this activation function. As *x* increases along the positive *x*-axis, the Mish function rises continuously, effectively avoiding the saturation issues often associated with capped functions. This unbounded increase aids in maintaining a robust gradient flow, crucial for the learning process in neural networks. Conversely, as *x* moves along the negative *x*-axis, the output of the Mish function gradually tends toward zero but does not exhibit the abrupt zero boundary characteristic of the ReLU function. This gradual approach to zero allows Mish to maintain a smoother gradient flow, especially for slightly negative values, enhancing the network’s ability to learn from a wider range of input data without the limitations imposed by the hard zero cut-off found in ReLU. This feature of the Mish function makes it a valuable asset in deep learning models, contributing to improved learning dynamics and overall network performance.

### 3.4. Data Augmentation

**Figure 4:**
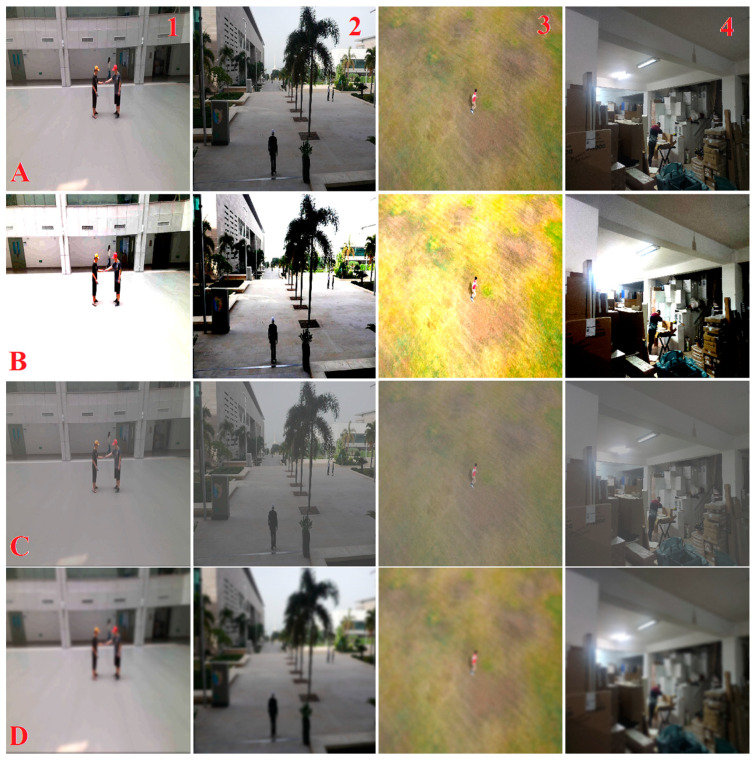
UAVHuman Dataset (1), UAV123 Dataset (2–3), Own Collection (4). Original image (**A**), increased brightness (**B**), decreased contrast (**C**), and blurred (**D**).

In this study, the emphasis on enhancing the dataset’s quality and diversity through targeted data augmentation techniques is critical for the model’s performance. By employing adjustments in brightness and contrast, along with the strategic application of blur effects, the aim is to create a dataset that better represents the variability and challenges of real-world scenarios. These augmentations are critical in training models for indoor detection detections. They address unique indoor challenges like varying lighting conditions, complex background clutter, and diverse spatial layouts. Brightness and contrast adjustments help in simulating different lighting scenarios, an essential aspect of indoor environments. The other technique used for augmentation is blur effects. It is particularly useful in simulate the effect of motion or focus variations that the UAV camera might encounter. These data augmentation techniques enrich the dataset, making it more reflective of real-world indoor conditions. This upgraded dataset ensures that the model can generalize better across various indoor environments, significantly enhancing its performance in detecting humans under different conditions. Data augmentation, as an intelligent approach, is crucial for the development of a robust, adaptable, and accurate indoor human detection system for UAV applications. An augmentation example for the IHD dataset is shown in Figure 4. The details of the datasets and their usages are discussed later in the Section 4.

## 4. Experiments and Evaluation

### 4.1. Datasets and Preprocess

**Figure 5:**
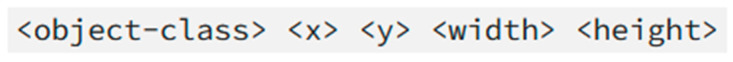
YOLO format annotation. Object class stands for the desired object’s ID; x and y are the center of the labeled bounding box (BB); width and height are the size of the BB.

This study encountered many complex environments for indoor object detection, specifically for human detection. Some of complex cases are low-light conditions, blurry input (source camera), close-up objects, very far objects, different human postures, and mixed human conditions. To overcome these scenarios, the focus was placed on the quality of the dataset. For this reason, in the training set, four datasets were used in combination. For distant objects, the VisDrone dataset [67] was used, for medium-sized objects, the UAV123 dataset [68] was used, for indoor human objects, the UAVHuman dataset [69] was used, and for some complex environments like low-light, blurry scenes, different positions of human, and different indoor clutter a specially collected dataset was used. To prepare the datasets for training, their annotations were converted to YOLO’s label txt format (Figure 5) and split into train, validation, and test directories. In each directory, there were ‘images’ and ‘labels’ subdirectories for training. Table 2 represents the distribution of the datasets, including a special mixed dataset (IHD dataset) designed for optimal training in complex environments.

#### 4.1.1. VisDrone Dataset

**Figure 6:**
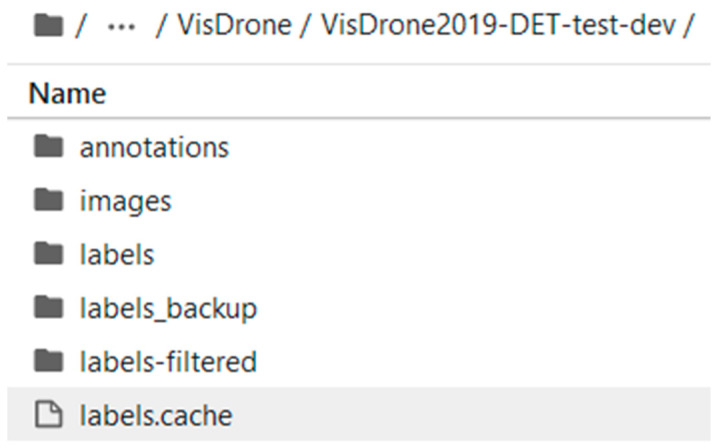
Converted annotations for VisDrone test-set directory.

The VisDrone dataset, developed for drone-based image analysis, stands out for its extensive collection of images and video sequences captured from various types of drones. This dataset is particularly significant for the development and evaluation of object detection on aerial vehicles due to its diverse range of urban and rural landscapes, different weather conditions, and a wide variety of objects and scenarios. The VisDrone dataset includes challenges like small-object detection, a high density of objects, and complex background variations, making it a robust testing ground for a model’s detection capabilities. The dataset’s high variability in object sizes and its emphasis on aerial perspectives provide a unique opportunity for enhancing the model’s accuracy and efficiency in drone-based surveillance and monitoring applications. In Figure 6, the converted and prepared test set of the VisDrone dataset directory is shown. The dataset comprises ten object classes, including pedestrian, people, bicycle, car, van, truck, tricycle, awning-tricycle, bus, and motor. The dataset’s key contribution for this study is the collection of small-sized human objects captured from a distant aerial perspective.

#### 4.1.2. UAVHuman Dataset

UAVHuman is a comprehensive dataset focusing on human detection and activity recognition from aerial perspectives. This dataset is critical for the advancement of indoor human detection applications related to indoor scenes, human-based actions, and human surveillance. UAVHuman encompasses a wide range of human activities and postures captured under different environmental conditions, offering a rich source for training and testing human detection algorithms. The dataset’s diversity in the scale, orientation, and density of human subjects presents a significant challenge for prediction models to maintain high accuracy and reliability, especially in complex and dynamic scenarios. Incorporating UAVHuman into training significantly enhances the model’s ability to accurately detect and track humans from aerial viewpoints, which is essential for effective drone-based human surveillance. In this dataset specifically, “ActionRecognition” video sets are used and processed for training. These videos captured from various indoor conditions and different human poses. Not only RGB videos but also Fisheye videos are used for enhancing the dataset. The key contribution of the dataset for this study is providing large-sized human objects from an indoor aerial view.

#### 4.1.3. UAV123 Dataset

The UAV123 dataset, specifically designed for object tracking from aerial platforms, provides a unique dimension to human model development. This dataset includes a variety of scenarios where objects are captured in different environmental conditions, altitudes, and speeds. The relevance of the UAV123 dataset to human detection lies in its rich collection of dynamic sequences, which are instrumental in improving the model’s tracking accuracy and robustness in real-world applications. The challenges presented by UAV123, including rapid object movement, varying scales, and occlusions, are crucial for the development of an effective human detection model. Training and evaluating on the UAV123 dataset not only enhance model’s performance in object tracking tasks but also ensures its adaptability and effectiveness in diverse aerial surveillance contexts. The key contribution of the dataset for this study is providing medium-sized human objects from an aerial view.

#### 4.1.4. Authors’ Own Dataset

**Figure 7:**
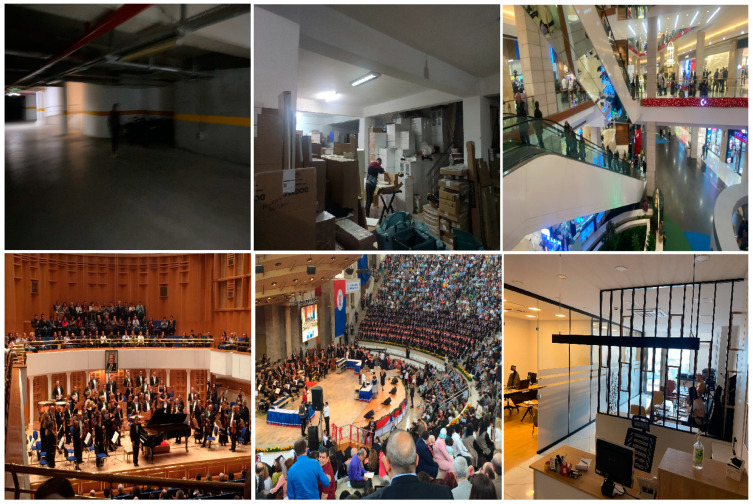
Collection of authors’ own dataset. Pictures from blurred low-light indoor garage, cluttered warehouse, higher view shopping mall, different light conditions auditorium, concert hall, and work office.

State-of-the-art datasets for aerial object detection primarily focus on strong, generic scenarios. However, when it comes to specific aspects like low-light environments, different altitudes, and blurry indoor conditions, these datasets may not suffice for the optimal training and detection performance of indoor human detection models. For this reason, this study utilizes a combination of three existing datasets and the authors’ own specially collected dataset. This specially collected dataset has been put together from various complex public indoor areas, including caves, stadiums, shopping malls, warehouses, and offices. The primary goal is to enrich the authors’ own dataset with diverse scenes, thereby training the proposed model to handle extreme real-world indoor scenarios effectively. Additionally, the dataset includes frames with blurred images to simulate the real-time vibrations experienced by drones in motion. Many real-time applications struggle with blurry pixels and poor-quality resolutions in object detection. One of the most challenging aspects in human detection is discerning the posture of the human body. To address this challenge, images capturing different human postures and angles in indoor conditions have been included. Figure 7 displays specific views and an indoor collection from the dataset.

The construction of the authors’ own dataset, as shown in Table 3, reflects an inclusive design, focusing on training an indoor human detection model for effective adaptation across a broad spectrum of real-life indoor scenarios. It includes 2278 images, categorized by area, sample count, object distance (far, normal, or close), and lighting conditions (bright, normal, or dark). The dataset consists of various scenes from working offices, malls, warehouses, caves, stadiums, garages, and auditoriums. The Working Office category includes 373 images and focuses on an emphasis on ‘normal’ and ‘close’ distances, which are crucial for systems requiring precision in confined spaces. Lighting conditions are well distributed, providing data for systems to learn from at different times of the working day. In the Shopping Mall category, which includes 622 images, the dataset captures a broad range of distances, featuring ‘far’ and ‘normal’ categories that align with the vast spatial layouts of such environments. The lack of images under ‘dark’ lighting conditions indicates a focus on the more prevalent lighting scenarios encountered in real-life shopping malls. The Warehouse category includes 459 images, with a significant portion representing ‘far’ distances and ‘dark’ lighting, highlighting the challenging visibility conditions in such indoor areas. This can be instrumental for indoor scenarios to operate effectively in environments with low light. The Cave category, featuring 85 images from caves, focuses on ‘normal’ and ‘close’ distances, predominantly under ‘dark’ and ‘bright’ lighting conditions. This can be important for models that need to learn in environments with unpredictable lighting and shadows. In cave scenarios, the lighting conditions are typically dark or bright due to the projector lighting used inside. The Stadium category includes 108 images, predominantly featuring ‘bright’ lighting conditions, which reflect the typically well-lit scenarios associated with stadiums. The distribution of images across various distances suggests a varied range of subject positionings, from the camera to objects on the field. Garages, featuring 509 images, provide a crucial setting for identify humans in ‘normal’ to ‘dark’ lighting and across all distances. This is particularly important for ‘far’ and ‘normal’ distances, which are essential in security and surveillance applications. In the Auditorium category, 122 images focus on ‘bright’ and ‘normal’ lighting conditions, likely reflecting the controlled lighting scenarios typical during indoor events. The emphasis in this category is on crowded events in well-lit environments, spanning various distances.

Each scenario in the dataset is carefully chosen to reflect the complexity of indoor environments where drones must detect humans. The diversity in lighting and spacing ensures that the model trained on this dataset can generalize across a wide range of indoor settings, making the dataset a valuable asset for the development of sophisticated surveillance systems.

#### 4.1.5. IHD Dataset

The IHD dataset, built for indoor human detection from a UAV perspective, integrates four diverse datasets: VisDrone, UAVHuman, UAV123, and the specially collected authors’ own dataset. Its distribution comprises approximately 40% VisDrone, 20% UAV123, 28% UAVHuman, and 12% from the authors’ own collection. This blend is designed to cover a broad spectrum of UAV-based indoor human detection scenarios. The IHD dataset contains a total of 12,725 images. These images are divided into three parts for model training: 73% (9405 images) for the training set, 12% (1533 images) for the validation set, and 14% (1787 images) for the test set. In the process of merging data from various datasets, only those images that contained human classes were specifically included. This selective approach furnished us with a comprehensive view of human objects, covering a range of sizes from small to large, and showcasing various poses. Additionally, by combining data captured at different altitudes, a more effective representation of various indoor conditions was achieved.

In the preparation of the IHD dataset, various human object sizes and lighting conditions were considered, as shown in Table 4. The VisDrone dataset, containing 7482 images of human-class objects in day and night conditions, was used as a valuable source for small objects. Some images with unclear positions or poor quality were filtered out, resulting in 5090 images for the IHD dataset. The UAVHuman dataset was mostly used, with only 87 images filtered out due to poor quality. In the UAV123 dataset, 2545 images were selected out of 34,679 due to repetitive scenes and similar lighting conditions. Additionally, some data from the authors’ own dataset, such as shopping malls and offices with normal lighting and sizes, were filtered out because similar data were available in pretrained weight. The same filtering was applied to warehouse data with normal lighting and sizes. In total, 1527 images were used from the original 2278 images of the authors’ own dataset to ensure dataset quality and eliminate redundancy.

### 4.2. Evaluation Metrics

In the experiments to evaluate performance metrics, AP (average precision), F1 score, and accuracy were used. Accuracy is calculated as the ratio of true positives (TP) and true negatives (TN) to the total number of samples identified, as shown in Equation (3). Here, TP refers to correctly predicted positive instances, and TN refers to correctly predicted negative instances. It is important to note that false positives (FP) are instances incorrectly predicted as positive, and false negatives (FN) are actual positives that are incorrectly predicted as negative.

(3) $$\mathrm{Accuracy}=\frac{\mathrm{TP}+\mathrm{TN}}{\mathrm{TP}+\mathrm{FN}+\mathrm{FP}+\mathrm{TN}}$$

Precision and recall are two fundamental metrics used to evaluate the performance of classification models, especially in contexts where the balance between true positive and false positive predictions is crucial. Precision, as shown in Equation (4), measures the accuracy of the positive predictions made by the model. It is the ratio of true positives (TP) to the sum of true positives and false positives (FP). High precision indicates that the model is reliable in its positive predictions. Recall, as shown in Equation (5), on the other hand, measures the model’s ability to detect true positives from all actual positive instances. It is calculated as the ratio of true positives to the sum of true positives and false negatives (FN). High recall signifies that the model is effective in capturing a high proportion of actual positive cases. Both metrics are crucial for understanding a model’s effectiveness, with precision being more focused on the correctness of positive predictions, and recall on the completeness of capturing positive cases.

(4) $$\mathrm{Precision}=\frac{\mathrm{TP}}{\mathrm{TP}+\mathrm{FP}}$$

(5) $$\mathrm{Recall}=\frac{\mathrm{TP}}{\mathrm{TP}+\mathrm{FN}}$$

Average precision (AP), as shown in Equation (6), measures the model’s ability to classify objects correctly and rank them based on the confidence of predictions, often summarized as mean Average precision (mAP) across multiple classes. The F1 score, as shown Equation (7), serving as the harmonic mean of precision and recall, provides a single metric that balances both the false positives and false negatives, crucial in scenarios where each type of error has significant implications or in the presence of imbalanced datasets. Intersection over Union (IoU), as shown in Equation (8), quantifies the accuracy of object localization by computing the ratio of the overlap area to the union area between predicted (*B_D_*) and ground truth bounding boxes (BGT). Together, these metrics offer a comprehensive evaluation of an object detection model’s capabilities, spanning both classification accuracy and the precision of object localization, allowing for a nuanced understanding and comparison of different models in the field.

(6) $$\mathrm{AP}=\int_{0}^{1}p\left(r\right)dr$$

(7) $$F1\;\mathrm{Score}=2*\frac{\mathrm{Precision}*\mathrm{Recall}}{\mathrm{Precision}+\mathrm{Recall}}$$

(8) $$\mathrm{IoU}=\frac{|B_{D}\cap B_{GT}|}{\left|B_{D}\cup B_{GT}\right|}$$

In evaluating the model’s efficiency for real-time applications, the focus was on two critical performance metrics: GFLOPS (giga-floating-point operations per second) and FPS (frames per second). GFLOPS provided a measure of the computational power, indicating how many billion floating-point operations the model could handle per second, while FPS is the model’s ability to process and render frames in a timely manner. These metrics provided a thorough insight into the model’s efficiency, highlighting its ability to meet high-speed computational demands and performance in real-time environments.

### 4.3. Training Parameters and Environment

In this study, the proposed model was trained and evaluated on both the VisDrone and IHD datasets. The number of epochs and the batch size were set to 200 and 16, respectively. The SGD optimizer was used for training and the initial learning rate was set to 0.01. The training environment for the experiments was as follows: Intel(R) Xeon(R) Gold 6258R CPU@2.70 GHz, 512 GB of RAM, NVIDIA Tesla V100 32 GB XM2 GPU, CUDA version 11.8, Pytorch 2.0.1 + cu118, and TorchVision 0.15.2 + cu118.

Before training the model, it is crucial to define evaluation metrics and initialize training parameters. For this purpose, the chosen parameters were mean average precision at Intersection over Union (IoU) 0.5 (mAP0.5), precision, recall, frames per second (fps), model parameter count, and giga-floating-point operations per second (GFLOPS), with specific settings detailed in Table 5. Setting hyperparameters is vital for effecting model performance and the success of algorithmic improvements. In enhancing the YOLOv7-tiny model, maintaining consistent hyperparameter settings is essential. This consistency ensures the effectiveness of model improvements and enables accurate performance comparisons before and after enhancements. Modifying hyperparameters while enhancing the algorithm might affect the ability to determine if performance variations are a result of the algorithm’s inherent improvements or are due to changes in the hyperparameters. Thus, to ensure a transparent and accurate evaluation of the advancements, this paper maintains a steady set of hyperparameters.

### 4.4. Results

#### 4.4.1. Ablation Study

In order to verify the effectivity of YOLO-IHD, ablation studies were conducted. To ensure accuracy and performance, all experiments were conducted using the same parameters and environments. Adaptive non-maximum suppression (Adaptive NMS) [70] dynamically adjusts the overlap threshold for object detections, improving accuracy in crowded scenes by reducing false negatives. It balances precision and false alarms by varying the threshold based on object density, making it particularly useful for detecting closely spaced objects. The Adaptive NMS (non-maximum suppression) algorithm is employed to manage redundant detection frames, with the NMS threshold value set at 0.5. When the Intersection over Union (IoU) values of two detection boxes fall below 0.5, any unnecessary detection boxes are efficiently suppressed following the standard NMS algorithm.

As shown in Table 6, the incremental contributions of different modifications to the baseline YOLOv7-tiny model were systematically dissected and analyzed. The baseline YOLOv7-tiny model achieved an mAP@0.5 of 35.20%, with precision, recall, and F1 score metrics providing a foundational understanding of its performance. The first adaptation, labeled A1, introduced a small-object detection layer to the baseline model. This enhancement resulted in a notable improvement across all metrics, with mAP@0.5 increasing to 47.23% and corresponding increases in precision, recall, and F1 score, reflecting the model’s enhanced capability to recognize smaller objects. Building on A1, adaptation A2 integrated the cross stage partial spatial pyramid pooling fast (CSPSPPF) technique. This further elevated performance, with mAP@0.5 reaching 50.05%. The CSPSPPF method significantly boosted the model’s feature extraction and fusion capabilities, as evidenced by the increased precision and recall values. Adaptation A3 extended A2 by incorporating augmented data, simulating a variety of indoor conditions to improve the model’s robustness and generalization. This extension led to a substantial leap in performance, with mAP@0.5 surging to 70.95%. The inclusion of diverse training examples translated to marked improvements in model precision, recall, and F1 score. The culmination of these adaptations resulted in the YOLO-IHD model, which additionally employed the Mish activation function. YOLO-IHD achieved the highest mAP@0.5 of 77.71% in the experiments, along with the highest precision, recall, and F1 score, underscoring the effectiveness of the Mish function in enhancing model learning dynamics and overall performance. This ablation study clearly demonstrates the individual and collective benefits of each methodological enhancement, culminating in a robust YOLO-IHD model that sets a new standard for performance in the evaluated tasks. In summary, YOLO-IHD is composed of an added small-object detection layer, the CSPSPPF module, extensive data augmentation, and the Mish activation function.

**Figure 8:**
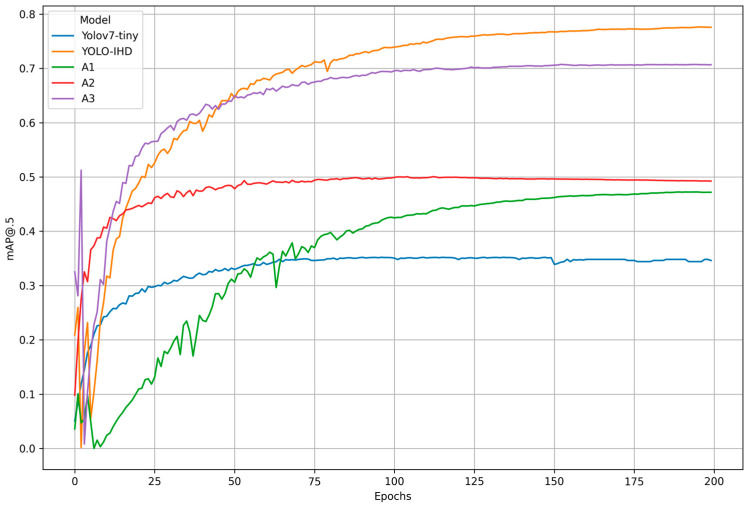
Incremental impact of different enhancements on the YOLOv7-tiny model. The table compares the baseline performance with successive integrations of a small-object detection layer (A1), the CSPSPPF module (A2), data augmentation techniques (A3), and the Mish activation function, which resulted in the best model, YOLO-IHD. Performance metrics include mAP@0.5, precision, recall, and F1 score on the IHD dataset.

The incremental improvements in mAP@0.5 through successive enhancements during the training of the YOLO-IHD model are shown in Figure 8.

#### 4.4.2. Comparison with Other YOLO Algorithms

In this study, the performance of the developed YOLO-IHD model was benchmarked against other YOLO models using the VisDrone dataset, which is widely recognized for its complexity and real-world applicability in aerial surveillance scenarios. During this comparative analysis, the focus was specifically on the average precision (AP) metric for human detection (Pedestrian and People classes). This targeted approach means that only the human detection values were carefully considered, providing a direct and relevant comparison of each model’s ability to recognize and track human figures within the dataset’s diverse aerial images. By isolating this metric, it was ensured that the comparison accurately reflected each model’s proficiency in the critical task of human detection, thereby demonstrating the YOLO-IHD model’s capabilities in a focused and precise manner.

In the comparative analysis of object detection models, the proposed YOLO-IHD model exhibited substantial advancements over current state-of-the-art models, as proved by the results presented in Table 7 from the VisDrone dataset. YOLO-IHD achieved an impressive 69.55% mAP@0.5 for human detection, markedly outperforming other models in the ‘Pedestrian’ and ‘People’ categories. For instance, compared to the earlier versions such as YOLOv3, which scored 12.8% and 7.8%, respectively, for ‘Pedestrian’ and ‘People’, and YOLOv7-tiny with 36.5% and 34.4%, YOLO-IHD showed substantial improvements. Even when measured against more recent models like YOLOv7, PDWT-YOLO, and YOLOv8, which had mAP@0.5 scores ranging from 44.25% to 45.15%, the proposed YOLO-IHD model stood out with a significantly higher accuracy. Moreover, YOLO-IHD surpassed the performance of MS-YOLOv7, which already presented a robust detection capability with 63.2% and 51.7% for ‘Pedestrian’ and ‘People’, respectively. The consistent AP across both ‘Pedestrian’ and ‘People’ for YOLO-IHD underscores its reliability and the efficacy of its integrated improvements, such as the added small-object detection layer, the CSPSPPF module, data augmentation, and Mish activation function, specifically tailored for the challenges of aerial human detection.

#### 4.4.3. Comparison with State-of-the-Art Algorithms

This section presents a comprehensive comparison of the proposed YOLO-IHD model with other leading state-of-the-art (SOTA) algorithms in the domain of indoor human detection, as presented in Table 8. The evaluation is based on several key performance metrics: the number of parameters (Params), the computational complexity as measured by GFLOPs, the frames per second (FPS) indicating real-time processing capability, and the mean average precision at an Intersection over Union (IoU) threshold of 0.5 (mAP@0.5). The evaluation of the YOLO-IHD model against other state-of-the-art algorithms was conducted using the IHD dataset and processed on an NVIDIA Tesla V100 GPU. This rigorous testing environment ensures a fair comparison in terms of performance and efficiency.

The YOLO-IHD model, consisting of 6.86 million parameters, demonstrated a commendable balance between model complexity and detection efficacy. With a computational demand of 18.82 GFLOPs, it managed to sustain a high frame rate of 186.6 FPS, indicative of its real-time applicability. The proposed model outperformed the other methods particularly in precision, achieving a mean average precision (mAP) of 77.71% at an IoU threshold of 0.5, substantially surpassing its counterparts. The YOLOv7-tiny, while possessing a similar parameter count of 6.21 M and a higher frame rate of 256.4 FPS, fell behind significantly in detection precision, with a mAP of 35.20%. Despite its lower computational load of 81.1 GFLOPs and moderate frame rate of 75.6 FPS, the SSD also achieved a mAP of 31.43%. The Faster-RCNN, with a much larger model size of 137.1 M parameters and a higher computational requirement of 246.2 GFLOPs, did not leverage its complexity to improve precision, achieving a mAP of 33.21% and a lower frame rate of 18.1 FPS. CenterNet, although it features a higher frame rate than Faster-RCNN at 29.4 FPS, similarly did not match the YOLO-IHD in precision, achieving 34.82%. The standard YOLOv7 model, which has a parameter count of 36.2 million and requires 106 GFLOPs, achieved a mAP of 54.61% at a reduced frame rate of 129.8 FPS. This underscores YOLO-IHD’s superior precision performance, as it delivers a significant improvement in detection accuracy with fewer parameters and a competitive processing speed.

In summary, YOLO-IHD stands out as an optimized model for indoor human detection, especially for drone applications where real-time processing and high detection accuracy are important. It demonstrates superiority in achieving a fine balance between speed, accuracy, and computational efficiency, thereby leading the way for future research and applications in UAV-based indoor surveillance systems.

#### 4.4.4. Realtime Studies

To comprehensively evaluate the YOLO-IHD model’s efficiency, real-time tests were carried out using a drone as a mobile platform. These tests were critical in evaluating the model’s performance in real-world conditions that drones typically encounter, such as varying altitudes, angles, and lighting situations within indoor environments. The model was tested on two edge computing devices, Jetson Xavier NX and Jetson Nano, each chosen for their balance of computational power and form factor suitability for drone integration. In Table 9, the specifications of the edge devices are shown. During these tests, the frames per second (FPS) at diverse resolutions were measured to mirror the operational demands of drones, which often require processing high-resolution inputs to ensure precise detection and tracking. A drone’s capability to process such resolutions at a high FPS is crucial for applications requiring fine-grained detail and accuracy, such as search-and-rescue operations or indoor surveillance.

**Figure 9:**
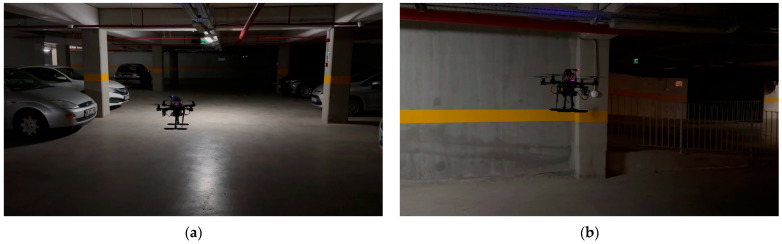
The custom-built drone is specifically designed for indoor experiments and testing the YOLO-IHD model. In the configuration presented, the drone utilizes an NVIDIA Jetson Nano, which is dedicated to testing and optimizing the indoor model. This setup ensures precise data collection and real-time processing, essential for the accurate evaluation and enhancement of the YOLO-IHD’s performance in indoor environments. (**a**) Flight in an indoor garage environment; (**b**) side view of drone.

The experimental hardware setup, as shown in Figure 9, involved a specially designed drone, which is equipped with two camera systems for real-time model testing: a D435i depth camera and a IMX477 MIPI (Mobile Industry Processor Interface) RGB camera. The depth camera operates at a resolution of 1280 × 720 at 30 fps, offering a field of view of 80 degrees horizontally and 40 degrees vertically, and it is effective over a range of 0.1 to 15 m. Its power efficiency is notable, requiring only 1.5 watts. The MIPI camera, on the other hand, stands out as a high-resolution camera, adept at capturing high-frame-rate video. Its resolution spans a broad spectrum, from VGA (640 × 480) to an impressive 12.3 MP (4056 × 3040), with frame rates ranging from 20 to 60 frames per second. This camera is recognized for its minimal power consumption, generally between 0.1 to 1 watt, and its compact form factor, with size and weight varying based on the model. A 4S 6000 mAh LiPo battery, ensuring extended operational capacity, powered the drone. Additionally, it incorporated an NVIDIA Jetson Nano for onboard data processing and a RPLiDAR system for effective obstacle detection and avoidance. The synergy of a high-capacity battery, robust processing capabilities, and advanced imaging technology renders the drone exceptionally suitable for comprehensive data gathering and diverse experimental applications. This configuration underscores the drone’s versatility and effectiveness in various research and testing scenarios for indoor environments.

Furthermore, the tests included flying the drone in dimly lit areas to specifically evaluate the model’s performance in low-light conditions—a common challenge in indoor surveillance. The YOLO-IHD model’s robustness was put to the test, and it demonstrated significant competence in detecting humans with high reliability, which is pivotal for ensuring safety and operational success. As a result, the real-time drone tests not only affirmed the YOLO-IHD model’s superior detection capabilities but also provided insights into the optimal operational parameters for drones employing this model. These results have significant implications for the deployment of drones equipped with advanced object detection systems, opening avenues for safer and more efficient indoor navigation and surveillance. 

When integrating custom models like YOLO-IHD with edge devices, direct deployment is often not feasible due to hardware constraints. Custom models typically require re-parameterization to match the computational capabilities of the target device. The process involves converting the model to an ONNX (open neural network exchange) format, which standardizes the model architecture. Once in ONNX format, TensorRT can then be applied to optimize the model, creating a highly efficient inference engine tailored for the edge device. This optimization includes layer fusion, precision calibration, kernel auto-tuning, and dynamic tensor memory, allowing the model to run effectively within the device’s resource limitations. These steps are crucial for leveraging the full potential of edge computing in real-time applications, ensuring that the balance between speed and accuracy is optimized for the specific use case like onboard human detection on drones.

The real-time analysis of the YOLO-IHD model, integrated with TensorRT across different quantization levels (FP32, FP16, and INT8), reveals a trade-off between detection speed and accuracy, as shown in Table 10. The model retains a consistent mAP of 76.23% with FP32 and FP16 precision on the NVIDIA Tesla V100 GPU, while the frame rate doubles from 185 FPS to 352 FPS when shifting from FP32 to FP16. This increase in speed can be attributed to the reduced precision (floating-point), which allows for faster computation without sacrificing accuracy. However, when the model is quantized to INT8, there is a noticeable decrease in mAP to 58.84%, but the speed increases significantly to 485 FPS on the V100. This suggests that for applications where speed is critical and some loss in accuracy is acceptable, INT8 quantization could be beneficial. On edge device hardware like the NVIDIA Jetson Nano and Xavier NX, the trade-offs are more pronounced. The higher frame rates observed on the Xavier NX, achieving 45 FPS with FP16 and 68 FPS with INT8 precision, underscore its viability for deployment in edge computing, reflecting its robust computational capabilities. Also, the Jetson Nano shows 27 FPS using FP16 precision and does not support INT8 due to hardware limitations. These results suggest that the highly optimized custom model can run efficiently on edge devices, ensuring efficient real-time performance even with reduced computational resources.

Table 11 compares the frames per second (FPS) performance of the YOLO-IHD model across different input resolutions and hardware configurations. For the 640 × 640 input resolution, the Nano achieves 8 FPS, which increases to 27 FPS with TensorRT-FP16. The Xavier NX starts at 25 FPS and improves to 45 FPS with TensorRT-FP16. At the lower resolution of 416 × 416, the Nano’s performance jumps from 12 FPS to 35 FPS with TensorRT-FP16, while the Xavier NX sees a rise from 34 FPS to 57 FPS.

The performance enhancement with the TensorRT-FP16 optimization on the Jetson Nano and Xavier NX is significant, especially on the Xavier NX, which shows a remarkable increase in FPS, nearly doubling at a resolution of 640 × 640. At the lower resolution of 416 × 416, the improvement remains substantial. This underscores the effectiveness of TensorRT optimization in achieving higher processing speeds necessary for real-time applications on edge devices. The results of these experiments inform the hardware selection process for drones, emphasizing the importance of choosing an edge device that offers both optimal real-time performance and meets the necessary resolution requirements.

In conclusion, integrating the YOLO-IHD model with TensorRT present certain limitations and challenges, particularly in adapting high-accuracy models for real-time applications. Employing various quantization levels introduces flexibility, allowing for customization in balancing speed and accuracy to fulfill the demands of real-time detection scenarios. As indicated in Table 9, the Jetson Nano, chosen for its low power consumption, offers significant benefits for drone applications. However, due to the Jetson Nano’s hardware limitations, the best balance between speed and accuracy is achieved using FP16 precision. This strategic choice underscores a specific trade-off, prioritizing efficient power usage while maintaining effective performance.

## 5. Discussion

In this study, the YOLO-IHD method was evaluated in various indoor environments like garages, shopping centers, and concert venues, focusing on detecting human subjects of different sizes. The results highlight the method’s effectiveness in diverse settings, emphasizing its significance for models used in densely populated indoor areas and its adaptability in challenging conditions.

In a comparison of object detection models, the proposed YOLO-IHD model demonstrates significant advancements over existing state-of-the-art models in the VisDrone dataset, as evidenced by the data in Table 7 and Table 8. YOLO-IHD achieves a remarkable 69.55% mAP@0.5 in human detection, substantially outperforming previous models in the ‘Pedestrian’ and ‘People’ categories. For example, it shows marked improvement over YOLOv3, which scored 12.8% and 7.8%, and YOLOv7-tiny, with 36.5% and 34.4%, in these categories. Even compared to recent models like YOLOv7, PDWT-YOLO, and YOLOv8, with scores between 44.25% to 45.15%, YOLO-IHD stands out for its higher accuracy. Furthermore, it surpasses MS-YOLOv7, a robust model with 63.2% and 51.7% in ‘Pedestrian’ and ‘People’. YOLO-IHD’s consistent AP in these categories highlights its reliability and effectiveness, attributed to its unique features like the added small-object detection layer, CSPSPPF module, data augmentation, and Mish activation function, specifically designed for aerial human detection.

**Figure 10:**
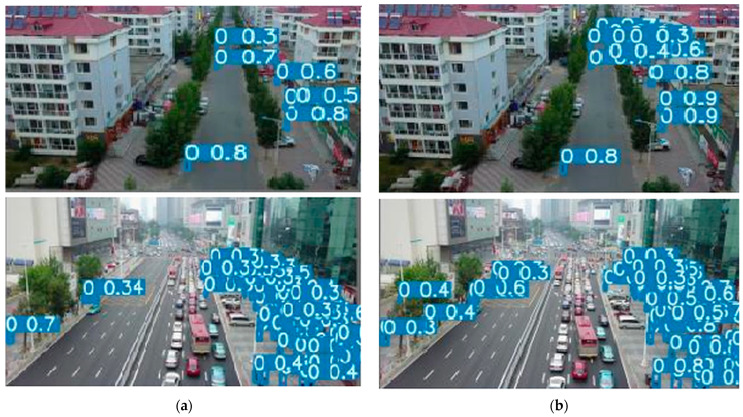
Detection results of VisDrone dataset: (**a**) prediction of baseline model YOLOv7-tiny; (**b**) prediction of YOLO-IHD model.

**Figure 11:**
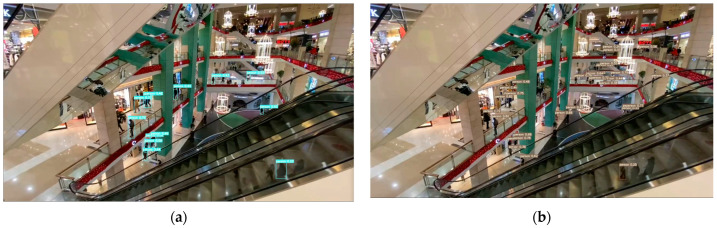
Human detection results of an indoor environment (Mall): (**a**) prediction of baseline model YOLOv7-tiny; (**b**) prediction of YOLO-IHD model.

The YOLO-IHD model was tested for its detection capabilities under different perspectives and indoor conditions. Figure 10 illustrates that, compared to the baseline model, YOLO-IHD detected more objects. The addition of the small-object detection layer to YOLO-IHD, as evident from the images, yielded successful results in detecting small objects. In Figure 11, the model successfully identified objects of varying sizes in a shopping mall under different angles and lighting conditions, outperforming the baseline model. This success can be attributed to the model being trained with an augmented dataset, which enhanced its prediction accuracy for objects of different sizes, angles, and views.

**Figure 12:**
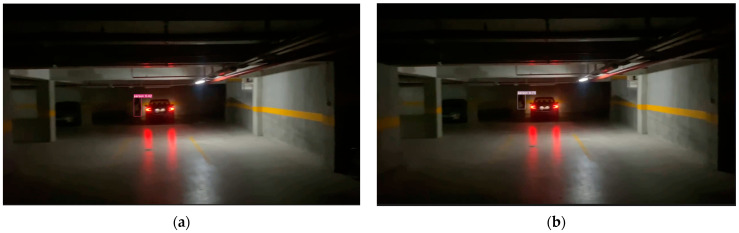
Human detection results of an indoor environment (Garage): (**a**) prediction of baseline model YOLOv7-tiny; (**b**) prediction of YOLO-IHD model.

**Figure 13:**
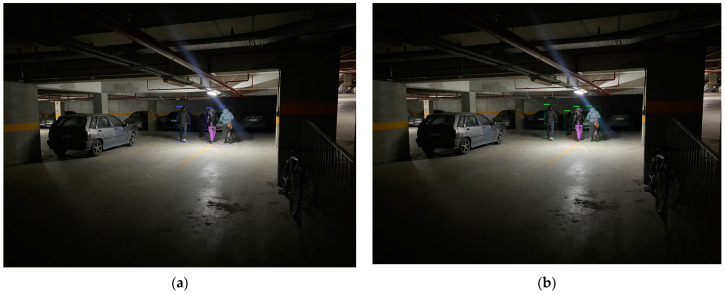
Human detection results of an indoor garage environment with multiple persons: (**a**) prediction of baseline model YOLOv7-tiny; (**b**) prediction of YOLO-IHD model.

**Figure 14:**
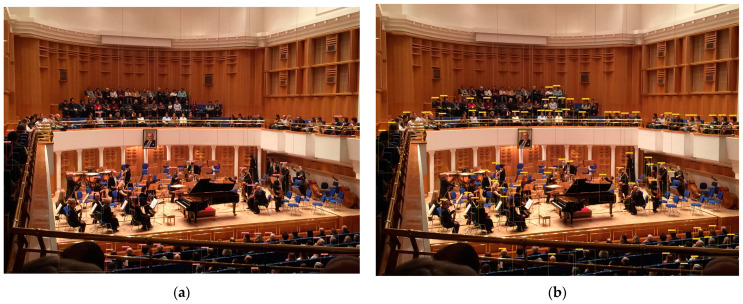
Human detection results of an indoor environment (Auditorium): (**a**) prediction of baseline model YOLOv7-tiny; (**b**) prediction of YOLO-IHD model.

Figure 12 presents a detection study conducted in a closed garage. The baseline model also managed to detect human objects. However, the primary reason for the 31% increase in accuracy of the YOLO-IHD model in darker areas is its enhancement with CSPSPPF and Mish functions. Additionally, a series of experiments were conducted involving multiple subjects in an enclosed garage environment. These experiments demonstrated that the YOLO-IHD model not only surpasses the baseline model in terms of accuracy but also exhibits superior detection capabilities. This is particularly evident in scenarios involving small-scale objects, as illustrated in Figure 13. In these instances, whereas the baseline model failed to detect a small human object, the YOLO-IHD model successfully detected it, underscoring its enhanced performance in complex low-light environments. In Figure 14, the model was tested in an auditorium, a setting characterized by a complex background and varying lighting conditions. This was among the most challenging experiments due to the combination of complex context and varying light. Despite these challenges, the YOLO-IHD model showed improved performance over the baseline model in this complex context.

The conditions and ambiance in Figure 14 are considered crucial for future development efforts. The ability of YOLO-IHD to perform well in such a challenging and dynamic environment highlights its potential for further enhancements and wider applications in real-world scenarios.

## 6. Conclusions

This study introduces significant advancements for indoor human detection methods using a refined version of the YOLOv7-tiny. The YOLO-IHD model incorporates specific modifications that substantially enhance its precision in human detection, making it particularly effective for drone-based applications. These modifications include the integration of a small-object detection layer, the adoption of the Mish activation function, and an enhanced spatial pyramid pooling (SPP) mechanism. Together, these enhancements have resulted in a robust model for the complexities of indoor environments and drone-specific challenges. Relative to the baseline YOLOv7-tiny model, YOLO-IHD has shown a significant improvement in performance, achieving a 42.51% increase in the IHD dataset and a 33.05% increase in the VisDrone dataset. This considerable enhancement in accuracy underscores the model’s sophisticated design and its alignment with practical human detection surveillance needs. The proposed model’s real-time applicability was evaluated on edge computing platforms, revealing that YOLO-IHD operates at 27 FPS on Jetson Nano and 45 FPS on Xavier NX. These frame rates are indicative of the model’s capacity to function efficiently in real-time applications, suggesting its suitability for deployment in edge devices where computational efficiency is important.

The YOLO-IHD model represents a substantial advancement in the field of real-time indoor human detection, offering a robust and well-tested solution that enhances the surveillance capabilities of drones. This research provides insights and developments poised to revolutionize autonomous surveillance in complex indoor scenarios. Its novel integration of a small-object detection layer, the utilization of the Mish activation function, and the enhancement of the spatial pyramid pooling mechanism demonstrate a significant improvement in current lightweight detection models. Furthermore, the quantization of the proposed model for onboard edge devices is noteworthy for its real-time detection capability. These unique features ensure exceptional accuracy in complex indoor environments, a critical aspect for real-world applications.

For future work, to enhance YOLO-IHD’s efficiency in crowded areas, integrating a multi-scale detection mechanism is proposed. This mechanism allows the model to distinguish between individuals in close proximity by using distinct spatial features at various scales. For low-light conditions, a dual-phase approach involving automatic image enhancement algorithms coupled with infrared spectrum analysis could be implemented, enabling the model to adaptively switch between visual and thermal imaging for optimal detection. In occlusion scenarios, leveraging the depth data from Intel D435i, a 3D reconstruction of the environment can be utilized in conjunction with 2D image data. This hybrid approach will enable the model to infer the presence of humans even when partially obscured, by reconstructing the likely shape and position of occluded parts based on the environmental context. This technical enhancement aims not only to improve accuracy in complex scenarios but also to expand the operational versatility of YOLO-IHD in varied indoor environments.

## Figures and Tables

**Table 1:** List of real-time UAV studies and their capabilities.

Sources	Study Area	Advantages	Disadvantages	Real-Time UAV
[27,28,29,30]	Indoor UAV Navigation Systems	Detailed insights into various indoor positioning and navigation systems.	Focuses less on real-time data processing and computational constraints of smaller drones like those equipped with companion computer.	No
[31,32,33,34]	Machine Learning in Drone Vision Systems	Demonstrates successful integration of vision algorithms in drone systems.	Overlooks challenges in running complex models on low resource hardware and outdoor environments.	Partial
[35,36,37,38,39,40,41,42,43,44]	Obstacle Avoidance and Human Detection in UAVs	Offers valuable insights into UAV-based obstacle avoidance, crucial for navigating indoor spaces. Extends to human detection techniques applicable to indoor environments, with a focus on accuracy and real-time processing. Emphasizes sensor integration for enhanced detection capabilities.	Related studies do not directly address human detection using YOLOv7 on resource-limited hardware. In addition, there is a lack of real-world testing in diverse and dynamic indoor environments.	Yes
[45,46,47,48]	UAV Performance Analysis	Accuracy and responsiveness analysis of UAV performance, including detection.	Rarely discuss challenges of running models like human detection in real-time on resource-limited hardware.	Lack of in-depth analysis of trade-offs between model complexity and real-time processing capabilities.

**Table 2:** The dataset distribution used in this study is as follows.

Dataset	Class	Train	Validate	Test	Total
VisDrone	10	6471	548	3190	10,209
UAVHuman	1 (Human)	2450	350	850	3650
UAV123	1 (Human)	21,756	3567	9356	34,679
Own Collection	1 (Human)	1647	205	426	2278
IHD Dataset	1 (Human)	9405	1533	1787	12,725

**Table 3:** Areas and environmental conditions in the data collection phase of the authors’ own dataset. The ‘Object Distance’ column indicates the sample count for the object distance in each area, and the ‘Lighting Condition’ column provides details of the lighting environments corresponding to the total count for each area.

Area	Sample Count	Object Distance			Lighting Condition		
		Far	Normal	Close	Bright	Normal	Dark
Office	373	-	170	203	100	221	52
Shopping Mall	622	377	245	-	163	459	-
Warehouse	459	265	194	-	-	157	302
Cave	85	-	50	35	45	-	40
Stadium	108	56	30	22	88	20	-
Garage	509	249	158	102	-	211	298
Auditorium	122	98	24	-	63	47	12
Total	2278	1045	871	362	459	1115	704

**Table 4:** IHD dataset distribution across various environmental and lighting features.

Dataset	Object Size	Environment	Lighting	Sample Size
VisDrone	Small	Outdoor	Day and Night	5090
UAVHuman	Large	Indoor	Normal–Dark	3563
UAV123	Medium	Outdoor	Day Light (Normal–Shadow)	2545
Authors’ Own Collection	Various Sizes	Indoor	Bright–Normal–Dark	1527
IHD Dataset	Mixed Size	Indoor/Outdoor	All Conditions	12,725

**Table 5:** Model training parameters.

Parameter	Value
Initial Learning Rate	0.01
Epochs	200
Batch Size	16
Optimizer	SGD
Weight Decay	0.0005
Momentum	0.937

**Table 6:** Results of ablation experiments with the IHD dataset.

Methods	mAP@0.5 (%)	Precision (%)	Recall (%)	F1 Score
YOLOv7-tiny	35.20	49.34	36.80	0.42
A1	47.23	63.59	42.35	0.50
A2	50.05	60.18	49.64	0.54
A3	70.95	76.92	64.17	0.69
YOLO-IHD	77.71	78.83	71.60	0.75

**Table 7:** Comparison of YOLO-IHD with other YOLO methods.

Methods	Pedestrian (%)	People (%)	mAP@0.5 (%)
YOLOv3 [71]	12.8	7.8	10.3
YOLOv7-tiny	36.5	34.4	35.45
YOLOv7	51.4	37.1	44.25
PDWT-YOLO [71]	48.7	41.6	45.15
YOLOv8 [72]	50.2	39.7	44.95
MS-YOLOv7 [72]	63.2	51.7	57.45
YOLO-IHD *	**69.55**	**69.55**	**69.55**

* The proposed model consists of a single class, defined as ‘Person’. Therefore, the model’s detection and prediction outcomes for the ‘People’ and ‘Pedestrian’ categories in the VisDrone dataset are identical.

**Table 8:** Comparison of YOLO-IHD with other SOTA methods.

Model	Params (M)	GFLOPs	FPS	mAP@0.5 (%)
YOLOv7-tiny	6.21	13.3	256.4	35.20
SSD	15.5	81.1	75.6	31.43
Faster-RCNN	137.1	246.2	18.1	33.21
CenterNet	52	109	29.4	34.82
YOLOv7	36.2	106	129.8	54.61
**YOLO-IHD**	**6.86**	**18.82**	**186.6**	**77.71**

**Table 9:** Edge device specifications.

Specs	Jetson Nano	Jetson Xavier NX
CPU	Quad-Core ARM^®^ Cortex	6-core NVIDIA Carmel
Memory	4 GB	8 GB
GPU Architecture	128-core Maxwell GPU	384 CUDA^®^ cores + 48 Tensor cores Volta GPU
Flops	512 GFLOPS (FP16)	21 TOPS
Power	10 W	20 W

**Table 10:** Real-time performance results of YOLO-IHD after optimization using different TensorRT conversions. The experimental results are conducted on the IHD validation set and the inference results are obtained after converting the models into TensorRT with different quantization levels.

Library	mAP@0.5	FPS (V100)	FPS (Nano)	FPS (Xavier NX)
PyTorch	76.23	185	8	25
FP32	76.23	187	7	25
FP16	76.23	352	27	45
INT8	58.84	485	-	68

**Table 11:** Real-time performance results of YOLO-IHD on edge devices at various input resolutions, with the device power mode set to ‘MAXN’ for maximum computational performance.

Input Size	Jetson Nano		Jetson Xavier NX	
	Pytorch	TensorRT	Pytorch	TensorRT
640 × 640	8 FPS	27 FPS	25 FPS	45 FPS
416 × 416	12 FPS	35 FPS	34 FPS	57 FPS

## Data Availability

The datasets used in this study are public. The VisDrone, UAVHuman, and UAV123 datasets can be accessed through their official links. Additionally, our dataset has been made available in our GitLab repository. The repository is accessible at the following URL: https://gitlab.com/gokhankucukayan/ihd-dataset (accessed on 23 December 2023).
